# Carboxylesterase1/Esterase-x Regulates Chylomicron Production in Mice

**DOI:** 10.1371/journal.pone.0049515

**Published:** 2012-11-07

**Authors:** Ariel D. Quiroga, Jihong Lian, Richard Lehner

**Affiliations:** 1 Group on Molecular and Cell Biology of Lipids, University of Alberta, Edmonton, Alberta, Canada; 2 Department of Pediatrics, University of Alberta, Edmonton, Alberta, Canada; 3 Department of Cell Biology, Mazankowski Alberta Heart Institute, University of Alberta, Edmonton, Alberta, Canada; University of Bari & Consorzio Mario Negri Sud, Italy

## Abstract

Elevated postprandial plasma triacylglycerol (TG) concentrations are commonly associated with obesity and the risk of cardiovascular disease. Dietary fat contributes to this condition through the production of chylomicrons. Carboxylesterases have been mainly studied for their role in drug metabolism, but recently they have been shown to participate in lipid metabolism; however, their role in intestinal lipid metabolism is unknown. Carboxylesterase1/esterase-x (Ces1/Es-x) deficient mice become obese, hyperlipidemic and develop hepatic steatosis even on standard chow diet. Here, we aimed to explore the role of Ces1/Es-x in intestinal lipid metabolism. Six-month old wild-type and Ces1/Es-x deficient mice were maintained on chow diet and intestinal lipid metabolism and plasma chylomicron clearance were analyzed. Along the intestine Ces1/Es-x protein is expressed only in proximal jejunum. Ablation of *Ces1/Es-x* expression results in postprandial hyperlipidemia due to increased secretion of chylomicrons. The secreted chylomicrons have aberrant protein composition, which results in their reduced clearance. In conclusion, Ces1/Es-x participates in the regulation of chylomicron assembly and secretion. Ces1/Es-x might act as a lipid sensor in enterocytes regulating chylomicron secretion rate. Ces1/Es-x might represent an attractive pharmacological target for the treatment of lipid abnormalities associated with obesity, insulin resistance and fatty liver disease.

## Introduction

Elevated fasting and postprandial plasma triacylglycerol (TG) concentrations are associated with obesity and the risk of cardiovascular disease [1], [2]. Absorption of dietary TG in the intestine is a multi-step process encompassing fat digestion by duodenal/jejunal lipases, uptake of the hydrolytic products into enterocytes, resynthesis of TG, packaging of TG into chylomicrons and secretion of chylomicrons into the lymph and eventually the systemic circulation. Carboxylesterase 1/Esterase-x (Ces1/Es-x), also annotated as Ces1g, is expressed only in the lipoprotein-producing organs, the liver and the small intestine [3]. In order to determine whether Ces1/Es-x plays a role in lipid metabolism we generated *Ces1/Es-x^−/−^* mice. We found that *Ces1/Es-x^−/−^* mice are obese and present with increased hepatic lipogenesis and with over secretion of hepatic apoB-containing very-low density lipoproteins (VLDL). Because of the regulatory role of Ces1/Es-x in hepatic lipid biosynthesis, storage and secretion, and because of the expression of Ces1/Es-x in the intestine we hypothesized that the enzyme might have a similar regulatory role in intestinal lipoprotein production and secretion. Here, we show that Ces1/Es-x senses the amount of fat present in the intestine and regulates its secretion.

## Materials and Methods

### Animals

Unless otherwise specified, 5–6 months old female mice were fed *ad libitum* a chow diet (LabDiet, PICO Laboratory Rodent Diet 20, 23.9% protein, 5% fat, 48.7% carbohydrates). Wild-type and Ces1/Es-x deficient mice were 25% of 129 and 75% of C57BL/6 background. The generation of *Ces1/Es-x^−/−^* mice is described elsewhere [3]. Mice, housed three to five per cage, were exposed to a 12 h light/dark cycle beginning with light at 8:00 a.m.

### Ethics Statement

All animal procedures were approved by the University of Alberta’s Animal Care and Use Committee and were in accordance with guidelines of the Canadian Council on Animal Care.

### Histology

Intestinal sections from the proximal 10 cm of the small intestine were collected from overnight fasted mice and embedded in paraffin for further histological analysis.

### Analysis of Postprandial Lipoprotein Secretion

After 12 h of fasting, mice received a bolus of 150 µl of olive oil by gavage. Blood from tail veins was withdrawn before (time 0) and at the indicated times after bolus (30, 60, 90, 180, and 240 min). Plasma TG levels were analyzed as described previously [4].

### Analysis of Chylomicron Clearance

Radiolabeled chylomicrons were prepared as previously described [5]. Animals were anesthetized and 100 µL of a chylomicron suspension isolated from plasma of the animals subjected to chylomicron secretion studies (see below) at time 120 min was injected through *vena cava*. Blood was collected from tail veins at 5, 10, 15 and 30 min after the injection, and radioactivity measured with a scintillation counter.

### *In vitro* Hydrolysis of Chylomicrons by Purified Lipoprotein Lipase

Radiolabeled chylomicrons from wild-type and *Ces1/Es-x^−/−^* mice were purified as previously described [5]. Chylomicron fraction was utilized for *in vitro* hydrolysis of chylomicrons by purified lipoprotein lipase (LpL). Briefly, hydrolysis of TG in chylomicrons was determined as the rate of release of unesterified fatty acid from TG. Chylomicrons and remnant negative controls (isolated from *ApoE^−/−^* mice [6]) were diluted in PBS containing 0.05% fatty acid-free BSA in order to reach the same amount of DPMs. Purified LpL (Sigma) was diluted in Krebs-Ringer-bicarbonate buffer pH 7.4 containing 0.05% fatty acid-free BSA at three different concentrations (2.5, 5 and 7.5 µg/assay). Reactions were performed according to Fielding and Fielding with slight modifications [7]. One hundred µL chylomicron/remnant fractions were incubated together with 100 µL LpL solutions for 10 min at 37°C. The reaction was stopped by the addition of 3.25 mL methanol/chloroform/heptane (1.41/1.25/1) followed by the addition of 1.05 mL K_2_CO_3_/Boric acid, pH 10. Radioactivity in the aqueous (top) phase was evaluated by scintillation counting.

### Analysis of Chylomicron Secretion

To determine lipoprotein secretion, blood was collected at time 0. Mice then received an intraperitoneal injection of 1 g poloxamer-407 (P-407, BASF Corp.) per kg of body weight and after 30 min, mice were *gavaged* with 150 µl of olive oil containing 10 µCi [^3^H]-triolein (Perkin Elmer). Blood was then collected from the tail vein after 60, 120, and 240 min. Plasma was prepared and lipids were extracted in chloroform:methanol (2∶1 v/v) containing lipid standards. Lipids were separated by thin-layer chromatography (TLC) on silica gel H TLC plates with the solvent system hexane:isopropyl ether:acetic acid (15:10:1 by vol). Lipids were visualized by exposure to iodine, recovered from the TLC plates and associated radioactivities were determined by scintillation counting (Beckman Coulter Inc.).

### Analysis of apoB48 Secretion Rate

Because overnight fasting strongly decreased the success rate of the cannulation procedure, mice were fasted for 2 h before cannulation and then kept anesthetized with isoflurane at 37°C for the length of the experiment. All cannulation procedures were performed before 12 pm [8]. Chylomicron proteins were resolved by SDS-PAGE followed by immunoblotting.

### Plasma Particle Size and Composition

Chylomicrons isolated from plasma at 120 min post gavage were suspended in 500 µl ice-cold PBS. A 1/50 dilution was made in water and the content was subjected to particle size analysis by dynamic light scattering on a Zetasizer Nano ZS (Malvern Instruments Ltd, UK) at 25°C.

### Isolation of Enterocytes and Labeling Studies

Enterocyte isolation, micelle preparation and enterocyte incubations were performed as previously described [9] with some modifications. Final concentration of the micellar components was: sodium cholate (0.14 mM), sodium deoxycholate (0.17 mM), phosphatidylcholine (0.17 mM), oleic acid (0.22 mM), monooleoylglycerol (0.19 mM) and 2 µCi/tube [^3^H]oleic acid. Cells were incubated with micelles for 2 h. Media were recovered and cells were washed twice with ice-cold PBS. Cells and media were subjected to lipid extraction and analysis as described above.

### Distribution of Dietary Fat Uptake

Uptake of dietary fat along the length of the small intestine was assessed as previously described [5]. Mice were fasted for 4 h and *gavaged* with 10 µCi of [^3^H]-triolein diluted in 100 µl of olive oil. Two hours later, the small intestine was excised, flushed with 0.5 mM sodium taurocholate in PBS and cut into 2-cm segments. Each segment was digested with 500 µl of 1 N NaOH at 65°C for 1 h, mixed with 5 ml scintillation liquid. DPMs were recorded with a scintillation counter.

### Stool Fat Analysis

Stool samples were collected after spontaneous defecation from 6-h fed animals after an overnight fast. Fat content in stools was analyzed by Sudan III staining [10]. Lipids were also semiquantitatively measured by extraction of the solid stools with organic solvents as described previously [11]. Finally, stool lipids were analyzed by TLC as described previously [12].

### mRNA Isolation and Real Time-PCR

Due to the circadian expression patterns of some genes (e.g. MTP), intestinal tissues were systematically harvested before 12 pm after 4 h fast. Total RNA was isolated from intestinal mucosa using Trizol reagent (Invitrogen). First-strand cDNA synthesis from 2 µg of total RNA was performed using Super-Script II reverse transcriptase (Invitrogen) primed by oligo(dT)_12–18_ primers. Real-time qPCR [Rotor-Gene 3000 instrument (Montreal Biotech, Canada)] was employed to detect other transcripts related to lipid absorption, lipogenesis and lipoprotein secretion using Platinum® Quantitation PCR Supermix (Invitrogen), SYBR Green I (Molecular Probes), and intron-spanning, gene-specific oligonucleotides (250 nM each primer) in a total volume of 25 µL. Primers for the genes analyzed in this paper are listed in Table 1.

**Table 1:** Primers used for quantitative real time PCR.

Gene	Sequence
Npc1l1	F:5′CATGGGCAGTGCGGTGTT T3’ R:5′CAGGAAGACCAGGCCGTGTAG3’
Abcg5	F:5′CTGCTCGCCTACGTGCTA3’ R:5′ATCTGGCAACTTCAGGATACAA3’
L-fabp	F:5′CAAAGTGGTCCGCAATGA3’ R:5′TAGACAATGTCGCCCAATG3’
Fatp4	F:5′GAAGGGGGACCAAGCCTA3’ R:5′AGTTCCTGGCACCTCAACAC3’
Fatp5	F:5′GTTCTCTGCCTCCCGATTCTG3’ R:5′TGGCCAAGCGCACTGTATGTA3’
Caveolin	F:5′GCATCTCAACGACGACGTG3’ R:5′ATGCCGAAGATCGTAGACAACA3’
Cd36	F:5′TGGCTAAATGAGACTGGGACC3’ R:5′ACATCACCACTCCAATCCCAAG3’
Abca1	F:5′TTGGATGGATTAGATTGGAC3’ R:5′ATGCCTGTGAACACGATG3’
Srebp-1c	F:5′ATGGATTGCACATTTGAAGAC3’ R:5′CTCTCAGGAGAGTTGGCACC3’
Apo-AIV	F:5′GTTTCAGAAGACGGATGTCACTC3’ R:5′CCTCTCAGTTTCCTGGGCTAGAT3’
Mttp	F:5′ATACATGCAAAATTGAGCGGTCT3’ R:5′CCTGGTCTCTTCTGCAAGCAC3’
Cyclophilin	F:5′TCCAAAGACGCAGAAAACTTTCG3’ R:5′TCTTCTTGCTGGTCTTGCCATTCC3’

### Immunoblotting

Except for analyses of intestinal Ces1/Es-x distribution (where all the intestinal segments were used) the proximal 10 cm of the small intestine have been employed for preparations of 20% homogenates for immunoblot studies. Intestinal homogenates were prepared in homogenization buffer (250 mM Sucrose, 20 mM Tris-HCl, 5 mM EDTA, pH 7.4) containing proteases inhibitors (Roche, Germany). Intestinal homogenate proteins were resolved by SDS-10% PAGE and transferred onto nitrocellulose membranes. Membranes were blocked with Tween-TBS containing 5% skim milk and probed with the corresponding primary antibodies, followed by appropriate HRP-linked secondary antibodies. Immunoreactive proteins were detected by enhanced chemiluminescence detection (GE Healthcare, UK). Plasma apolipoproteins and isolated chylomicron proteins were resolved by pre-casted gradient (4–15%) SDS-PAGE (BioRad, Hercules, CA), then the gels were treated as described above.

### Statistical Analysis

Data are presented as means ± SEM. Statistical significance was evaluated by unpaired two-tailed Student’s test. Time course studies were evaluated by two-way ANOVA followed by Bonferoni post-test (GraphPad PRISM® 4 software). P value <0.05 was interpreted as a significant difference.

## Results

### Intestinal Ces1/Es-x Protein Levels are Regulated by Nutritional Status

**Figure 1:**
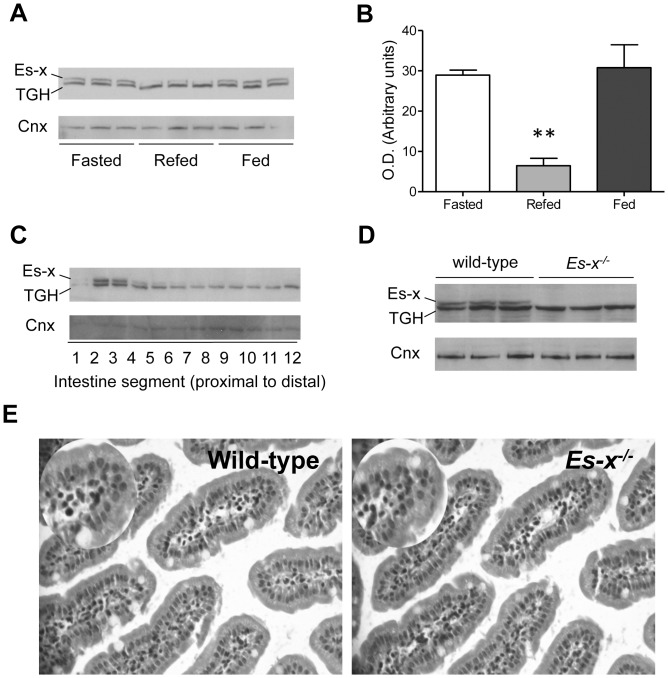
Intestinal Ces1/Es-x expression is regulated by nutritional status. (A) Intestinal Ces1/Es-x protein expression in different nutritional states. Mice were fasted for 24 h and refed for 6 h. Fasted and refed mice were euthanized at 8:00 P.M. TGH is a related carboxylesterase migrating at a lower M_r_ due to lesser glycosylation and is recognized by the polyclonal anti-Ces1/Es-x antibodies. Two µg intestinal proteins were subjected to analysis. Cnx, calnexin  =  loading control. (B) Quantitation of Ces1/Es-x immunoreactive bands obtained in different nutritional states. **p<0.01 Refed vs Fasted. (C) Ces1/Es-x protein distribution along the small intestine. Small intestine was cut into 12 pieces 2-cm long. Proteins were separated by SDS-PAGE and immunodetected with anti-Ces1/Es-x antibodies. Representative data from 3 different independent experiments are shown. (D) Absence of Ces1/Es-x protein (immunoblot) in the intestine from *Ces1/Es-x* ^−/−^ mice. (E) H&E staining of small intestine (200×) sections.

In wild-type mice, intestinal Ces1/Es-x protein levels were the highest after 24-h fast, and the lowest following 6-h re-feeding (Figure 1A and 1B), compared to the higher expression during the fasting and fed states. The faster migrating band is a carboxylesterase Ces3/TGH (also annotated as Ces1d) that shares over 70% of sequence similarity with Ces1/Es-x and is recognized by the anti-Ces1/Es-x polyclonal antibody.

### Ces1/Es-x Protein is Expressed only in Jejunum

Ces1/Es-x protein expression is restricted to the liver and the small intestine [3]. In the intestine Ces1/Es-x protein concentration is very low in the duodenum (segment 1) and is the highest in proximal jejunum (Figure 1C).

### Lack of Ces1/Es-x Protein Expression in *Ces1/Es-x^−/−^* Mice

To investigate the role of Ces1/Es-x in intestinal lipid metabolism, we generated global *Ces1/Es-x^−/−^* mice. Ces1/Es-x protein expression is completely absent in intestinal samples of *Ces1/Es-x^−/−^* mice (Figure 1D). Analysis from intestinal sections from wild-type and *Ces1/Es-x^−/−^* mice by haematoxylin-eosin (H&E) staining showed no morphological differences between the genotypes (Figure 1E).

### Impaired Chylomicron Clearance in *Ces1/Es-x^−/−^* Mice

**Figure 2:**
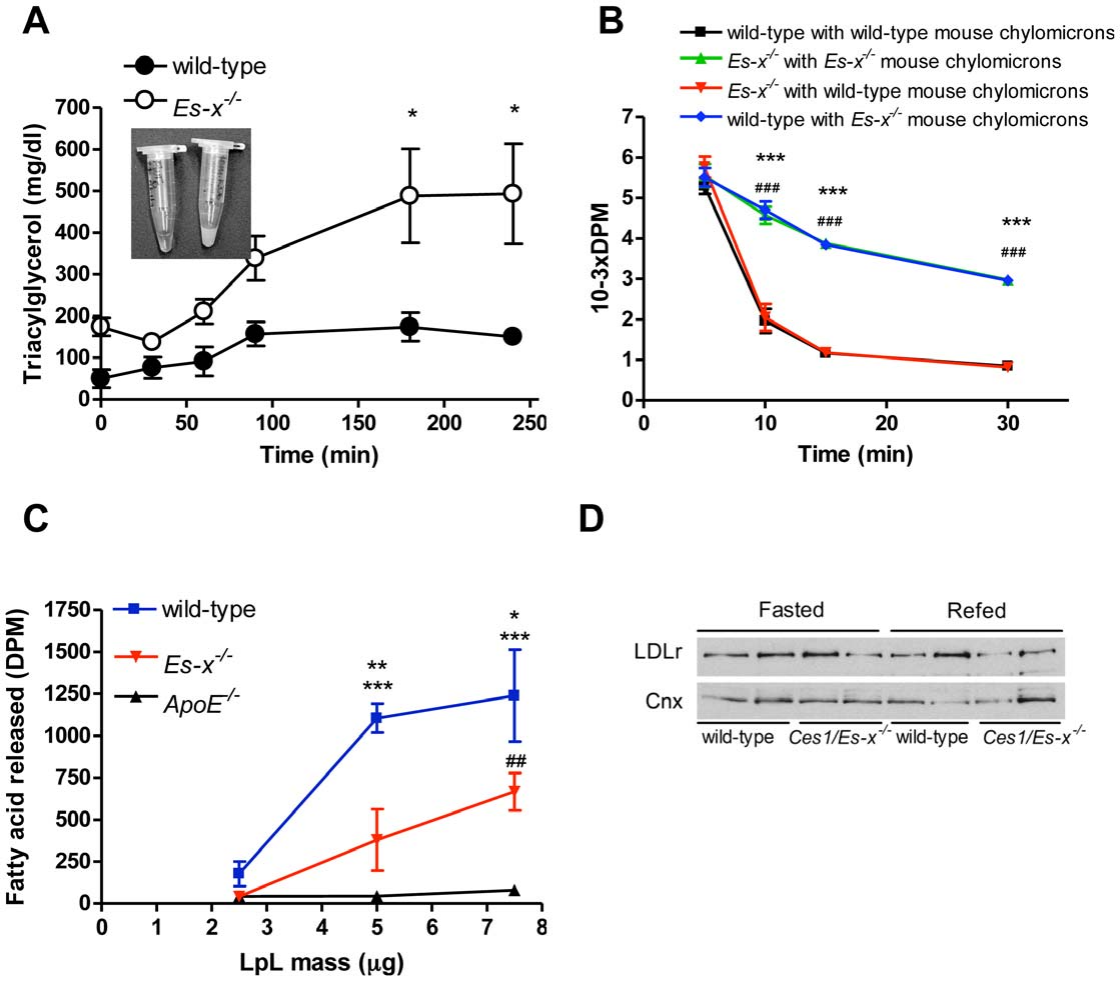
Impaired chylomicron clearance in *Ces1/Es-x^−/−^* mice. (A) Fat tolerance test in 12-h fasted mice. Mice were *gavaged* with 150 µl olive oil. Blood samples were collected at the indicated times, plasma was prepared and TG concentrations were measured by gas chromatography. N = 4–6 mice/group. Inset shows plasma appearance 4 h after the lipid load (wild-type on the left and *Ces1/Es-x^−/−^* on the right). *p<0.05 vs. wild-type. (B) Chylomicron clearance. Labeled chylomicrons were isolated from plasma of wild-type and *Ces1/Es-x^−/−^* mice and injected into abdominal veins of mice of both genotypes. Blood samples were collected, plasma prepared and radioactivity measured by scintillation counting. N = 6 mice/group. ***p<0.001 black line vs. blue line, ^###^p<0.001 green line vs. red line. (C) *In vitro* lipolysis of chylomicrons from wild-type, *Ces1/Es-x^−/−^* and *ApoE^−/−^* mice by isolated LpL. N = 3 mice/group. Details about the procedure are given in the main text. *p<0.05 vs. *Es-x* ^−/−^, **p<0.01 vs. *Es-x* ^−/−^; ***p<0.001 vs. *ApoE^−/−^,* ^##^p<0.01 vs. *ApoE^−/−^*. (D) Immunobotting showing hepatic LDLr protein expression from fasted and re-fed (6 h) mice.

After an oil bolus challenge, postprandial kinetics of fat absorption showed prolonged triglyceridemia with milky plasma appearance in *Ces1/Es-x^−/−^* mice (Figure 2A). Therefore, we investigated whether chylomicron clearance is affected in *Ces1/Es-x^−/−^* mice. Radiolabeled chylomicron particles isolated from both wild-type and *Ces1/Es-x^−/−^* mice were reciprocally administered into both wild-type and *Ces1/Es-x^−/−^* mice. Clearance of chylomicrons isolated from *Ces1/Es-x^−/−^* by wild-type mice was delayed when compared with clearance of either chylomicrons isolated from wild-type by wild-type mice, or chylomicrons from wild-type by *Ces1/Es-x^−/−^* mice (Figure 2B). Additionally, we also performed an experiment to evaluate lipolysis of lipoprotein particles by purified LpL. *In vitro* lipolysis of chylomicrons from *Ces1/Es-x^−/−^* mice was lower than that for wild-type chylomicrons when incubated with purified LpL (Figure 2C). Variations in expression levels of hepatic proteins such as low-density lipoprotein receptor (LDLr) can affect particle clearance [13], [14], [15], [16]. No changes in LDLr levels were observed between genotypes when evaluated by immunoblotting (Figure 2D).

### Abnormal Chylomicron Particle Composition in *Ces1/Es-x^−/−^* Mice

**Figure 3:**
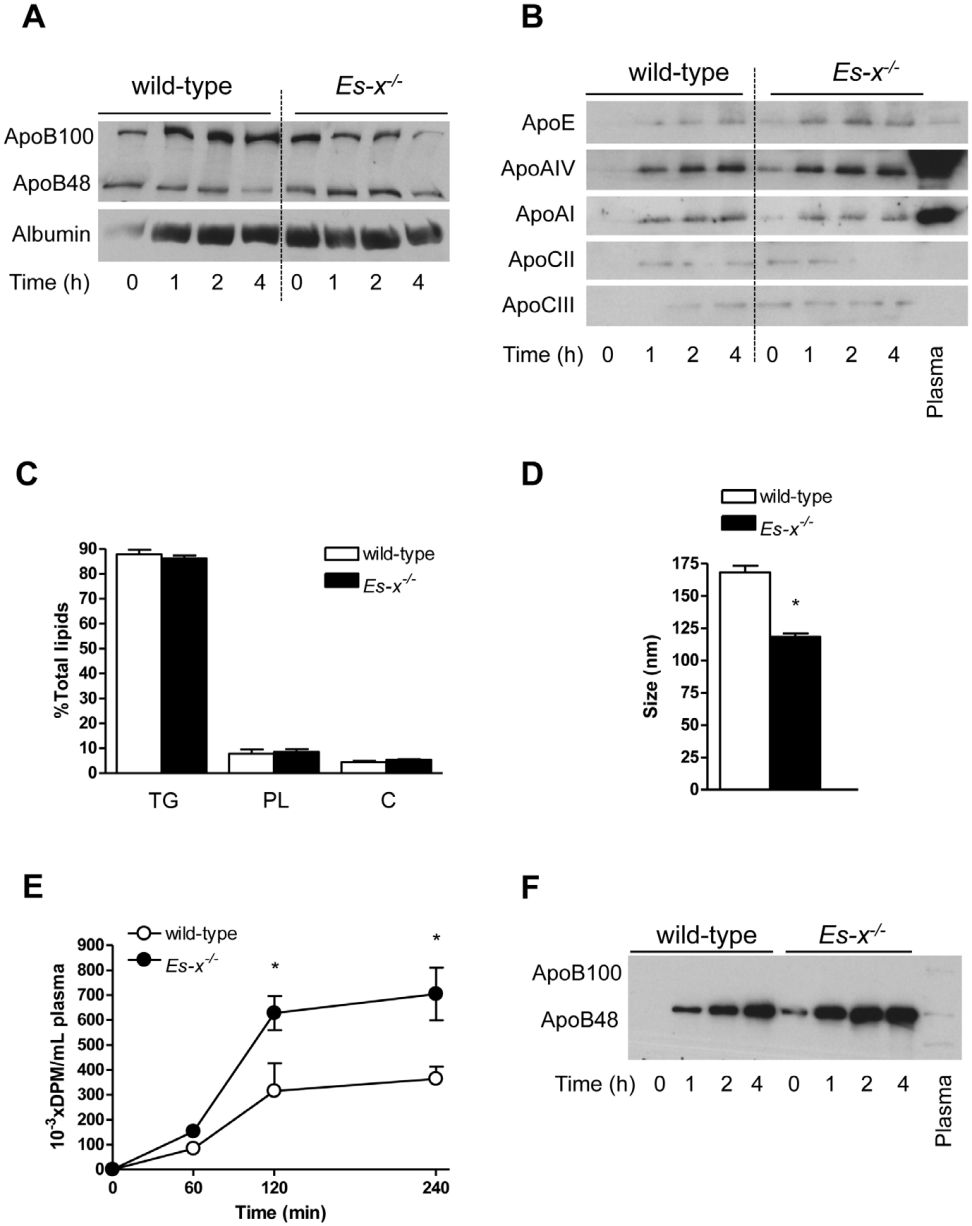
Chylomicrons from *Ces1/Es-x^−/−^* mice present with abnormal composition. (A) Immunoblotting showing plasma apolipoprotein B composition from mice injected with P-407. Plasma samples were prepared at the indicated times. Representative data from 3 independent experiments. (B) Immunoblotting showing apolipoprotein composition of chylomicrons isolated from wild-type and *Ces1/Es-x^−/−^* mice. Representative data from 3 independent experiments. (C) Chylomicron lipid composition. Chylomicrons were isolated lipids extracted and levels measured using commercial kits. N = 5 mice/group. PL, glycerophospholipids; C, total cholesterol. (D) Chylomicron size. Chylomicrons were isolated from wild-type and *Ces1/Es-x^−/−^* mice and size was evaluated by dynamic light scattering at 25°C. N = 5 mice/group, 5 measurements per sample. (E) Chylomicron secretion. Overnight fasted mice were injected with P-407, followed by an olive oil bolus containing radiolabeled triolein. Blood was collected at the indicated times and plasma prepared. Lipids were extracted, spotted onto TLC plates and resolved. Lipids were visualized by exposure to iodine, and radioactivity in TG was counted in a scintillation counter. N = 6 mice/group, *<p0.05 vs. wild-type. (F) Chylomicron apolipoprotein B secretion rate. Chylomicrons were collected through lymph duct cannulation. Proteins from 2 µL of lymph were resolved by SDS-5%PAGE and immunoblotted for apoB. Plasma control (1 µL) in the farthest right lane.

To address the potential mechanisms responsible for the delayed clearance of chylomicrons produced by *Ces1/Es-x^−/−^*, we determined plasma and/or chylomicron apolipoprotein composition. Increased abundance of apoB48 from time 0 up to 2 h post-gavage was observed in *Ces1/Es-x^−/−^* mice plasma (Figure 3A). The relative abundances of apoE, apoAIV and apoCIII were higher, while apoCII concentration was lower in isolated chylomicrons from *Ces1/Es-x^−/−^* mice in 4-h fasted mice (time 0) and following an oral fat challenge (at 1 to 4 h) compared to control wild-type mice (Figure 3B). No changes in the relative lipid composition were observed in the TG-rich lipoproteins (Figure 3C), which is in concert with significantly smaller TG-rich lipoprotein particles isolated from *Ces1/Es-x^−/−^* mice (Figure 3D). Altogether these results indicate an increased number of intestinal-derived (postprandial) apoB48 particles of smaller diameter in *Ces1/Es-x^−/−^* mice compared to lipoproteins produced by wild-type mice. Interestingly, the levels of plasma apoB100 (a VLDL marker) were lower in *Ces1/Es-x^−/−^* mice during the postprandial period (Figure 3A).

### Increased Chylomicron Secretion in *Ces1/Es-x^−/−^* Mice

To address chylomicron production rate, mice were injected with P-407, a detergent that inhibits LpL activity, followed by an oral bolus of olive oil containing radiolabeled triolein. *Ces1/Es-x^−/−^* mice secreted significantly more radioactivity incorporated into plasma TG compared to wild-type, indicative of increased secretion of chylomicron-associated TG (Figure 3E). To further investigate intestinal apoB secretion, chylomicrons were collected from lymph and apoB composition was determined by immunoblotting. As shown in Figure 3F, chylomicrons (apoB48) were present in the lymph of *Ces1/Es-x^−/−^* mice even in the absence of secretion stimuli (time 0). This increment in apoB48 secretion in *Ces1/Es-x^−/−^* mice was sustained up to 4 h after olive oil stimulus. These results are in line with hyperlipidemia observed in fasting *Ces1/Es-x^−/−^* mice [3].

### Enterocytes from *Ces1/Es-x^−/−^* Mice Accumulate Less Intracellular TG than Wild-type Enterocytes

**Figure 4:**
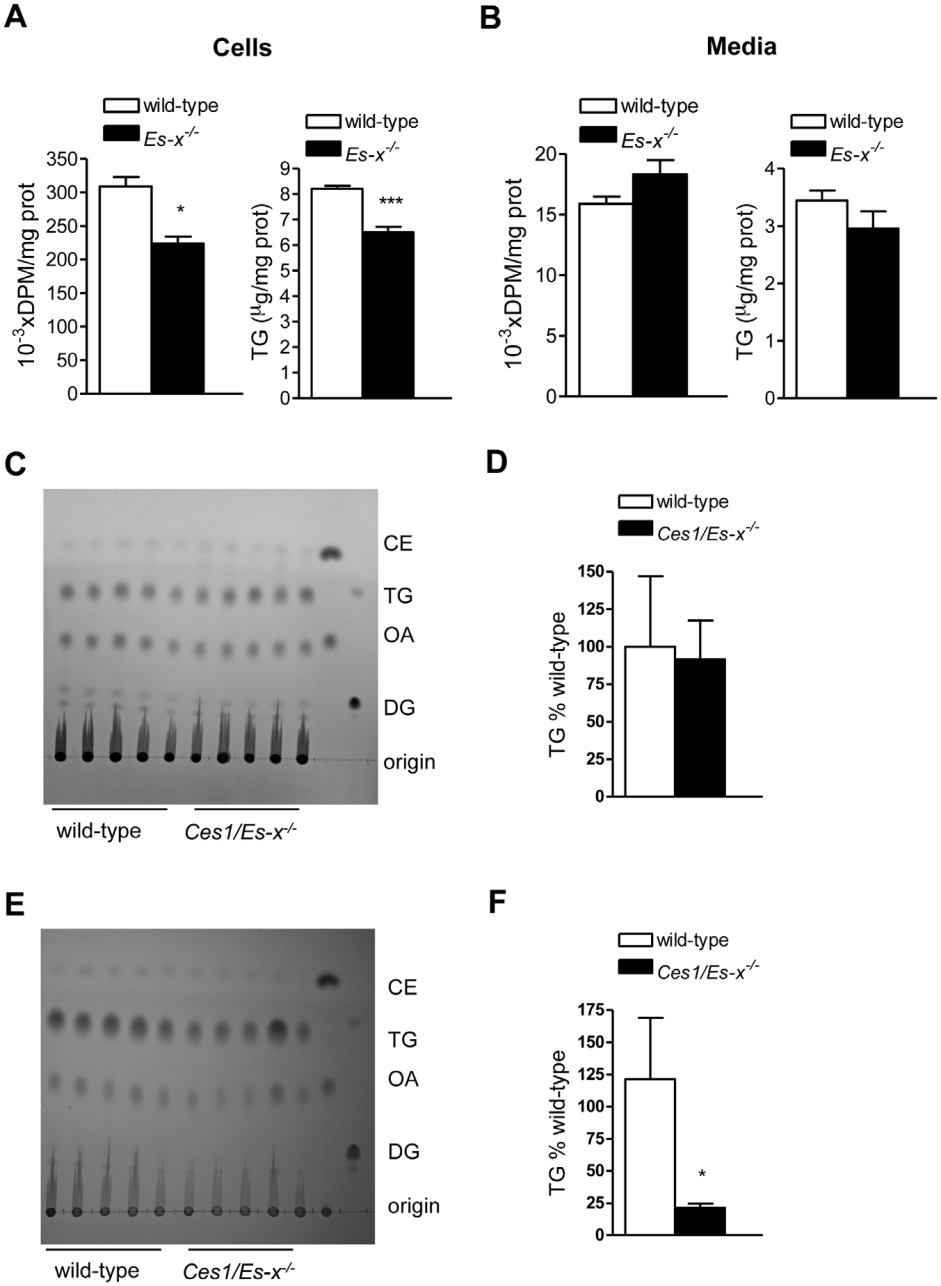
Lower TG accumulation in enterocytes from *Ces1/Es-x^−/−^* mice. (A) and (B) Enterocytes from fasted mice were isolated and incubated with micelles containing radiolabeled oleic acid. Cellular radiolabel incorporation into TG and media radiolabeled TG secretion were assessed by lipid extraction, TLC separation, iodine exposure and scintillation counting. Cellular and media TG mass was measured by gas chromatography. N = 4 mice/group, *p<0.05, ***p<0.001. (C) Lipid content in intestinal mucosal scrapings from mice fasted overnight and re-fed for 6 h. Lipid standards in the last two lanes on the right are: DG, diacylglycerol (dioleoylglycerol); OA, oleic acid, TG, triacylglycerol (triolein), CE, cholesteryl ester (cholesteryl oleate). (D) Quantitation of the TG band in (C). (E) Lipid content in intestinal mucosal scrapings from mice fasted overnight. Lipid standards in the last two lanes on the right are as in (C). (F) Quantitation of the TG band in (E). *p<0.05 vs. wild-type.

Enterocytes from *Ces1/Es-x^−/−^* mice accumulated 20% less intracellular TG than enterocytes from wild-type mice after 2 h incubation with micelles containing [^3^H]oleic acid (Figure 4A). No differences were observed in the mass or radioactivity in TG secreted to the media (Figure 4B). Interestingly, fat content in mucosal scrapings prepared from fed mice was similar for both genotypes (Figure 4C and 4D). However, during the fasted state fat content in mucosal scrapings from *Ces1/Es-x^−/−^* mice was significantly lower than in wild-type (Figure 4E and 4F).

**Figure 5:**
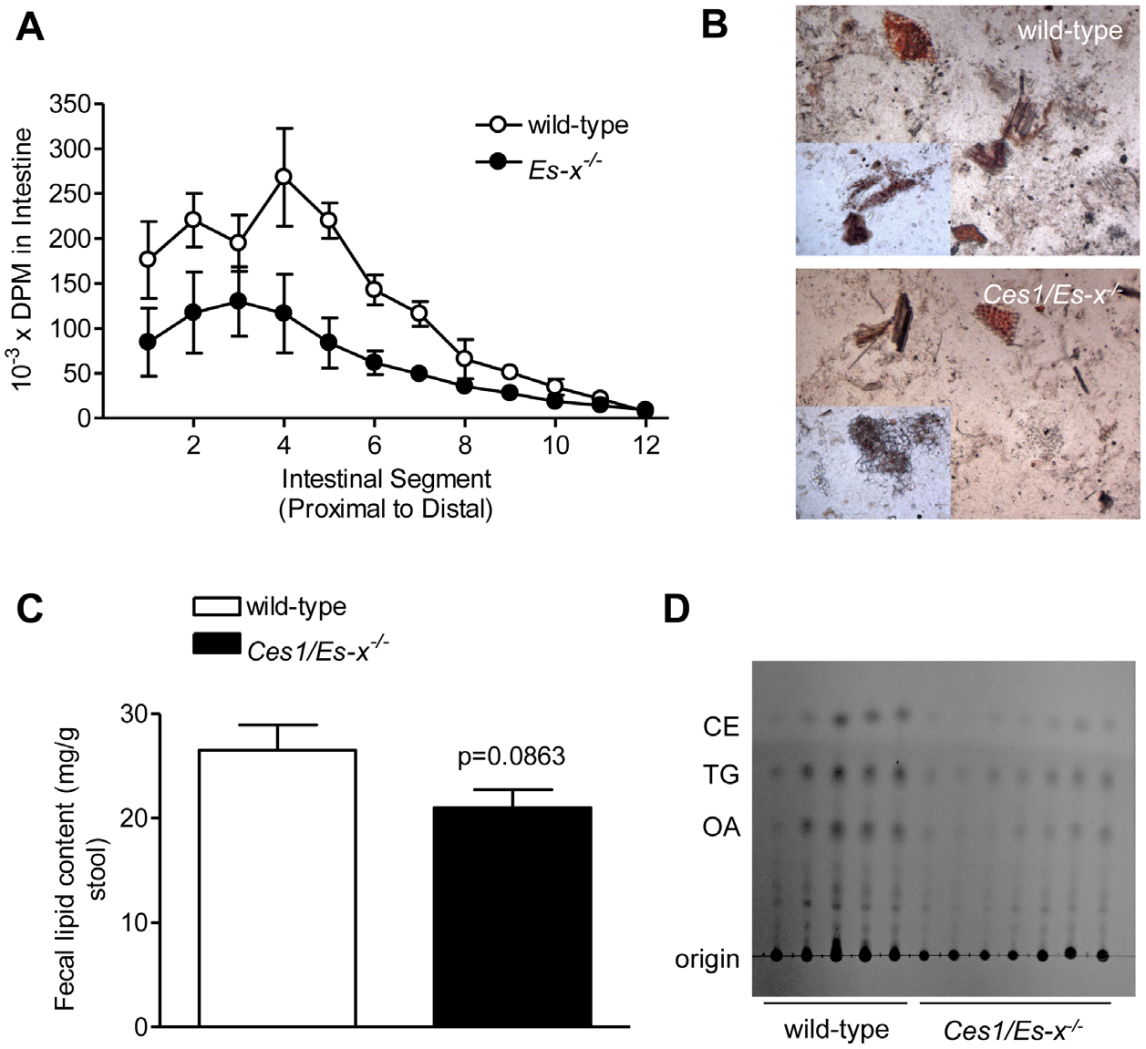
Decreased intestinal TG accumulation and absence of fat malabsorption in *Ces1/Es-x^−/−^* mice. (A) Uptake of dietary fat along the length of the small intestine. Mice were fasted for 4 h and gavaged with radiolabeled triolein diluted in olive oil. Two hours later, the small intestine was excised, flushed, cut, digested and radioactivity associated with the intestinal segments was determined. N = 3 mice/group. (B) Fat in stools. Sudan III staining of stool fat upon spontaneous defecation in fed mice. (C) Analysis of fat in stools by organic extraction after spontaneous defecation. N = 5–7 mice/group. (D) Fecal lipids analyzed by TLC. N = 5–7 mice/group.

We performed an *in vivo* assay to assess the uptake of dietary fat along the length of the small intestine. Intestines from *Ces1/Es-x^−/−^* mice accumulated significantly less radioactive TG along its length (Figure 5A); suggesting either decreased fat absorption or augmented incorporation into chylomicrons and secretion. Fat content in stools was not significantly different between the genotypes and was tending toward decrease in *Ces1/Es-x^−/−^* mice (Figure 5B–5D), indicating absence of fat malabsorption.

### Altered Expression of Lipid Metabolism Genes in *Ces1/Es-x^−/−^* Mice

**Figure 6:**
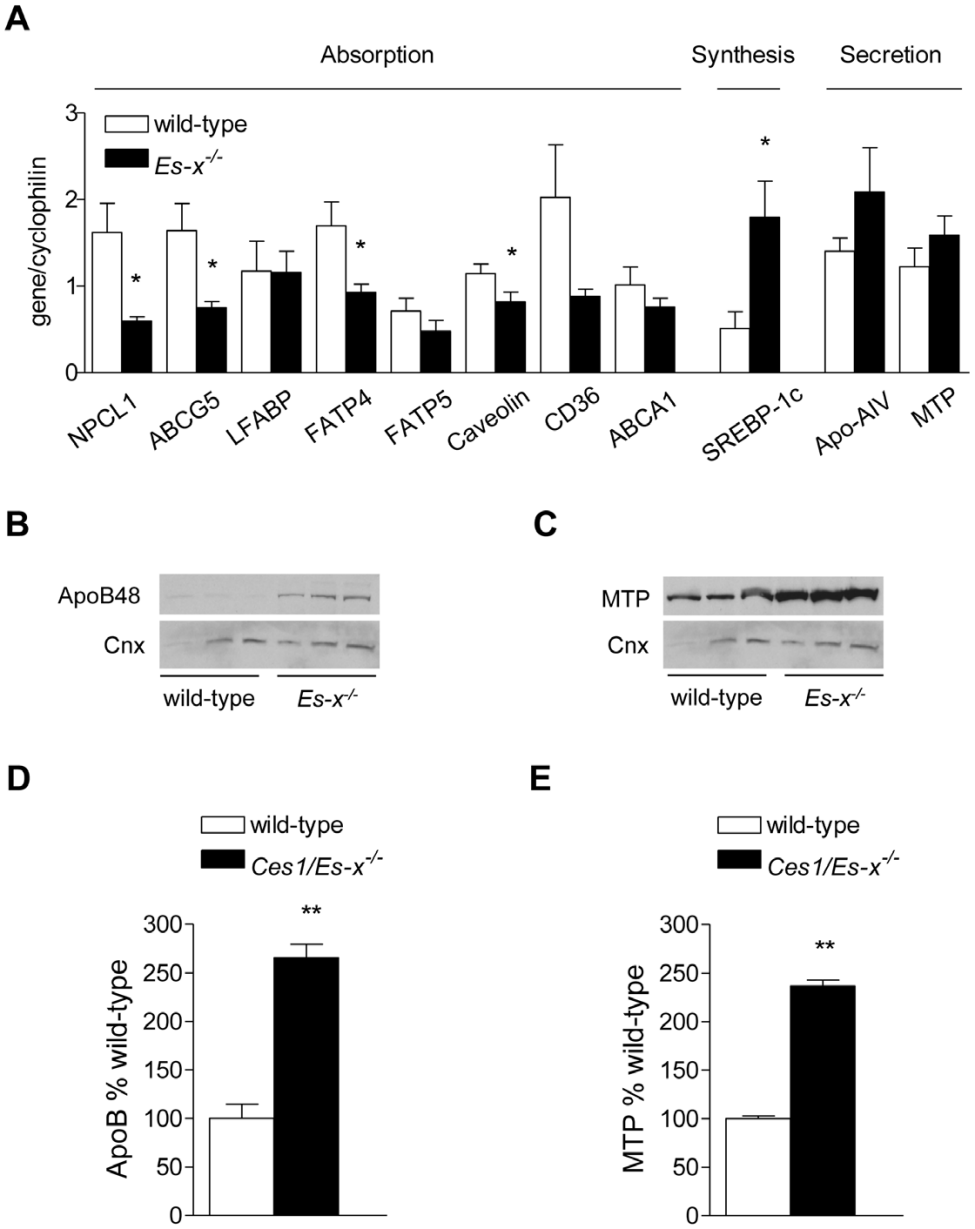
Decreased expression of lipid absorption and increased expression of lipid secretion markers in *Ces1/Es-x^−/−^* mice. (A) Intestinal expression of lipid absorption, synthesis and secretion genes in intestines from 24-week old mice was analyzed by qPCR. Mice were fasted for 4 h. N = 5 mice/group. *p<0.05 vs. wild-types. (B) Immunoblot of intestinal ApoB48. (D) Immunoblot of intestinal MTP. Same animals as in (A) were used. Cnx, calnexin  =  loading control. (D) and (E) quantitation of the immunoreactive ApoB48 and MTP bands, respectively. **p<0.01 vs. wild-type.

Despite the apparent absence of fat malabsorption gene expression analysis showed significantly decreased expression of genes involved in intestinal lipid absorption, such as NPC1L1, ABCG5, FATP4 and CD36 (Figure 6A). Interestingly, sterol regulatory element binding protein (SREBP)-1c expression was increased in *Ces1/Es-x^−/−^* intestine, but no changes in the expression in its target genes fatty acid synthase (FAS) or stearoyl-CoA desaturase (SCD)-1c were observed (not shown). The expression of genes involved in lipoprotein assembly Apo-AIV and microsomal TG transfer protein (MTP) was not different between the two genotypes. However, the protein expression of ApoB48 and MTP was significantly higher in intestinal homogenates from *Ces1/Es-x^−/−^* mice compared to wild-type (Figure 6B–6E).

## Discussion

Excessive TG storage in obese individuals is associated with serious health problems that include non-alcoholic fatty liver disease, insulin resistance, type 2 diabetes and cardiovascular disease. Detailed understanding of the regulation of TG metabolism in tissues is crucial for the development of new therapies for obesity-related disorders. The metabolism of TG is highly regulated from its initial absorption from diet, to its synthesis and storage in tissues, its release from the intestine and from the liver in lipoproteins and its hydrolysis to provide fatty acids for energy production. Plasma TG concentrations are determined by the rate of lipolysis over the rate of secretion of TG-rich lipoproteins such as chylomicrons and VLDL. We have shown that Ces3/TGH (Ces1d) participates in the assembly of VLDL by providing lipids for this process [4], [17] Another carboxylesterase, Ces1/Es-x (Ces1g), shares ∼76% protein sequence identity with Ces3/TGH including 100% amino acid sequence identity in the catalytic site. Ces1/Es-x is expressed only in the liver and proximal jejunum. Overexpression of Ces1/Es-x in McArdle RH-7777 (rat hepatoma) cells decreased TG accumulation and decreased VLDL secretion, suggesting that the carboxylesterase might negatively regulate TG storage [18]. In order to examine the contribution of Ces1/Es-x to lipid metabolism *in vivo,* we have generated Ces1/Es-x deficient mice. These mice are obese, hyperlipidemic, and present with impaired insulin sensitivity, increased hepatic lipogenesis, increased cellular lipid accumulation and increased VLDL secretion [3]. The role of carboxylesterases in intestinal lipid metabolism has not been investigated. In the present study we show that Ces1/Es-x actively participates in intestinal lipoprotein secretion impacting global lipid metabolism.

We have shown that hepatic Ces1/Es-x protein levels are decreased during fasting (a condition when VLDL secretion is high) [3]. When mice were re-fed after a 24 h fast, hepatic levels of Ces1/Es-x increased (attenuated VLDL secretion). Here we show opposite regulation of Ces1/Es-x levels in the intestine, where Ces1/Es-x protein expression is increased during the fasting period (low chylomicron secretion), and decreased after re-feeding (maximum chylomicron secretion). These results suggest that Ces1/Es-x might regulate the availability of substrates for lipoprotein assembly. Fat digestion, absorption and secretion have been shown to occur primarily in the proximal intestine [19]. High Ces1/Es-x expression in the proximal part of the small intestine is in line with the proposed role of this enzyme in the regulation of intestinal lipoprotein assembly.

*Ces1/Es-x^−/−^* mice presented with increased fasting apoB48. This strongly suggests the importance of the intestinal contribution to the characteristic hyperlipidemia in *Ces1/Es-x^−/−^* mice [3]. Furthermore, when these mice were challenged with an olive oil bolus increasing plasma TG concentrations were observed for up to 4 h suggesting either augmented chylomicron production, diminished clearance of the TG-rich lipoprotein particles, or a combination of both. We found that clearance of chylomicrons isolated from *Ces1/Es-x^−/−^* by wild-type mice was delayed when compared with clearance of either chylomicrons isolated from wild-type by wild-type mice, or chylomicrons from wild-type by *Ces1/Es-x^−/−^* mice. Two important conclusions can be drawn from these findings: i) the clearance capacity of lipoproteins by *Ces1/Es-x^−/−^* mice is not impaired and ii) chylomicron particles produced by *Ces1/Es-x^−/−^* mice might have abnormal composition that delays their catabolism. Indeed, after an oral lipid load, chylomicron abundance of apoE (and plasma, not shown) increased significantly in *Ces1/Es-x^−/−^* mice. It has been reported that high amounts of apoE inhibit LpL activity thereby increasing plasma TG levels [20], [21], although opposite effects have also been postulated [22]. Analyses of isolated chylomicron apoCs after an oral lipid load showed an increment in apoCIII (inhibitor of LpL) and a decrease in apoCII (activator of LpL) levels in *Ces1/Es-x^−/−^* mice, which may also contribute to the observed delay in chylomicron clearance. Both the size and number of particles regulate the metabolic fate of chylomicrons and remnant particles and are often associated with an increase in cardiovascular disease risk. While the number of particles appears to be more relevant than size with respect to particle clearance [23], [24], it has also been demonstrated that smaller particles of aberrant composition, might be cleared from plasma slower than larger particles [25], [26], [27], Because *Ces1/Es-x^−/−^* mice have normal clearance of lipoproteins isolated from control mice we conclude that the activity of LpL is normal in *Ces1/Es-x^−/−^* mice, and thus the increase in number, decrease in size and altered particle composition induced by the deficiency is responsible for the delayed clearance of TG-rich lipoproteins. In addition, we evaluated lipolysis of particles by purified LpL and showed that LpL-mediated lipolysis of chylomicrons from *Ces1/Es-x^−/−^* mice was diminished compared to chylomicrons isolated from wild-type mice. Altogether these results suggest normal activity of plasma lipases in *Ces1/Es-x^−/−^* mice and reinforce our conclusions that aberrant lipoprotein particle composition in *Ces1/Es-x^−/−^* mice is responsible for decreased particle clearance from plasma. Furthermore, hepatic protein expression of one of the key proteins involved in remnant clearance, LDLr, was similar in both genotypes.

*Ces1/Es-x^−/−^* mice present with decreased blood glucose concentration and increased insulin concentration [3]. Tso and colleagues recently showed that plasma apoAIV concentrations correlate with decreases in blood glucose concentration and transient increases in insulin secretion [28]. In line with this correlation, *Ces1/Es-x^−/−^* mice plasma apoAIV levels were increased in both fasting and postprandial states.

Interestingly, after a fat challenge *Ces1/Es-x^−/−^* mice accumulated significantly less TG along the length of the small intestine than wild-type mice. This is in agreement with increased chylomicron secretion, and with the pattern of Ces1/Es-x protein expression. In addition, cellular protein levels of ApoB48 and MTP, key factors in chylomicron production, were higher in *Ces1/Es-x^−/−^* intestine compared to wild-type in spite of similar mRNA expression of these genes, suggesting decreased degradation of these proteins. It was shown that inositol-requiring enzyme 1β (IRE1β) plays a key role in regulating MTP and in chylomicron production [29]. Interestingly, no differences were observed in IRE1β expression between genotypes in *Ces1/Es-x^−/−^* mice (not shown), suggesting that increased lipid availability might be responsible for the observed augmented chylomicron production. *Ces1/Es-x^−/−^* mice present with decreased intestinal lipid accumulation, which is accompanied by reduced expression of genes involved in lipid absorption, probably as a mechanism to compensate the hyperlipemic status in these mice. However, *Ces1/Es-x^−/−^* mice did not exhibit fat malabsorption.

These results clearly show that Ces1/Es-x is actively involved in the regulation of chylomicron assembly and secretion. The affected pathways in TG metabolism (trafficking, synthesis, chylomicron assembly and secretion, storage, lipolysis or oxidation) by Ces1/Es-x have not yet been fully elucidated. One possible mechanism is that Ces1/Es-x generates regulatory lipid signals within the endoplasmic reticulum of enterocytes that decrease TG secretion. It was shown in the liver that Ces1/Es-x releases regulatory polyunsaturated fatty acids (PUFA) from TG which in turn attenuate de novo lipogenesis via inhibition of processing of SREBPs. It is likely that the PUFA-specific-TG hydrolase activity of Ces1/Es-x plays a similar role in the intestine because intestinal expression of SREBP-1c is also induced in *Ces1/Es-x^−/−^* mice. Deregulated lipid synthesis, together with increased assembly, leads to augmented chylomicron secretion albeit with an aberrant apolipoprotein composition. This smaller aberrant particle reaches the circulation where it cannot be efficiently hydrolyzed by LpL, resulting in hyperlipidemia. To our knowledge, this is the first report about the role of a carboxylesterase in intestinal lipid metabolism and lipoprotein secretion. Our findings suggest that pharmacological interventions leading to augmented Ces1/Es-x activity might result in the alleviation of hyperlipidemia, obesity, insulin resistance and fatty liver disease. Future investigations will be directed to better understand the relative contributions of hepatic vs. intestinal Ces1/Es-x to lipid metabolism by using tissue specific knockout mice.
